# Zinc oxide-induced changes to sunscreen ingredient efficacy and toxicity under UV irradiation

**DOI:** 10.1007/s43630-021-00101-2

**Published:** 2021-10-14

**Authors:** Aurora L. Ginzburg, Richard S. Blackburn, Claudia Santillan, Lisa Truong, Robyn L. Tanguay, James E. Hutchison

**Affiliations:** 1 Department of Chemistry and Biochemistry, University of Oregon, Eugene, OR 97403 USA; 2 Sustainable Materials Research Group, School of Design, University of Leeds, Leeds, LS2 9JT UK; 3 Department of Environmental and Molecular Toxicology and the Sinnhuber Aquatic Research Laboratory, Oregon State University, Corvallis, OR 97333 USA

**Keywords:** Sunscreen, Zinc oxide, Photodegradation, Toxicity, Zebrafish, Formulation

## Abstract

Sunscreen safety and efficacy is generally evaluated based upon the properties of the individual chemicals in a formulation. However, the photostability of sunscreens has been shown to be highly dependent on the mixture of chemicals present. To better understand how sunscreen formulation influences stability, and to establish a foundation for probing the influence of zinc oxide additives, we formulated five different small-molecule based ultraviolet-filter (UV-filter) mixtures with a Sun Protection Factor (SPF) of 15. These mixtures contained active ingredients approved in either the United States or European Union and were designed to represent formulations of actual products on the market. We evaluated the photostability and toxicity of these mixtures in the absence and presence of zinc oxide after UV exposure for two hours. Changes in UV absorbance were minimal for all five small-molecule-based mixtures without zinc oxide. The presence of either micro- or nano-sized zinc oxide caused significant small-molecule photodegradation and the degraded mixtures exhibited higher levels of toxicity in embryonic zebrafish assays. This study suggests that caution must be taken when formulating sunscreens containing both zinc oxide and small-molecule UV-filters to avoid unintended consequences during use.

**Graphic abstract:**

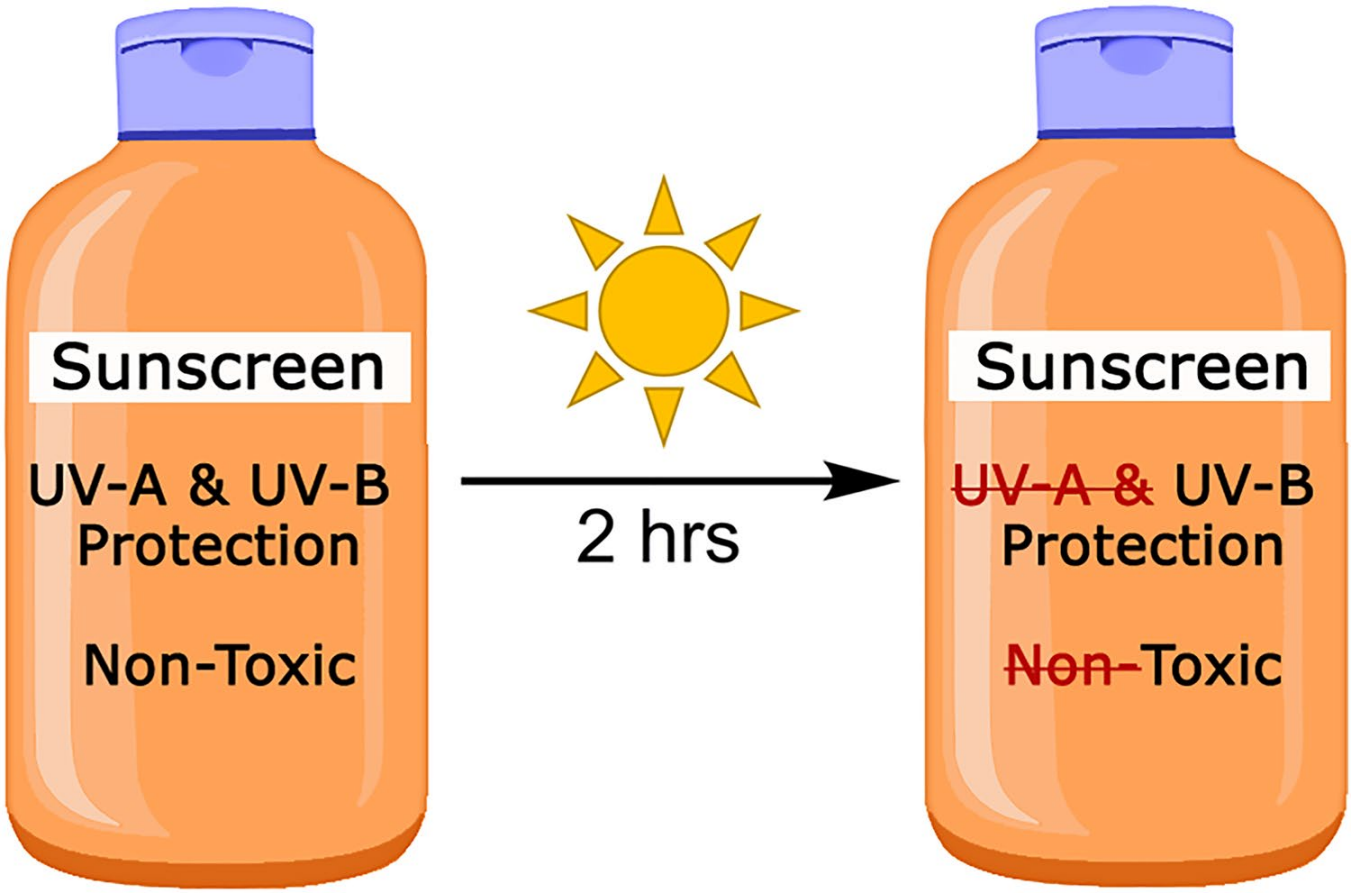

**Supplementary Information:**

The online version contains supplementary material available at 10.1007/s43630-021-00101-2.

## Introduction

Sunscreen efficacy and safety is of paramount importance for both human health [1] and the environment [2]. The limited list [3, 4] of chemicals available for use as sun protecting active ingredients is concerning, especially considering the emerging public scrutiny [2, 5–7] of ingredients. Within the past few years, there have been multiple highly publicized studies regarding the potential hazards of small-molecule based sunscreens on human health and aquatic environments [2, 5, 8, 9]. As of June 2021, the US Food and Drug Administration (FDA) sunscreen monograph listed only 16 ultraviolet-filters (UV-filters) (the active ingredients in sunscreens) approved for inclusion in cosmetic products. These include eight organic compounds that absorb primarily in the UV-B region (280–315 nm); four organic compounds that absorb in the UV-B and short-wave UV-A (315–340 nm) regions; but only two organic compounds that absorb primarily in the full (both short-wave and long-wave) UV-A region (315–400 nm) [3]. Filters that provide coverage of the UV-A region are particularly important because up to 95% of UV radiation reaching the Earth’s surface is UV-A [10, 11]. The FDA also approved the use of two inorganic “filters” that impede UV-A and UV-B transmission: titanium dioxide (TiO_2_) and zinc oxide (ZnO) [3]. ZnO and TiO_2_ are commonly employed to impede UV-A and UV-B transmission, respectively [12, 13].

Nomenclature surrounding UV-filter types is important, considering their scientific, commercial and popular usage. Sometimes small-molecule UV-filters have been described as “chemical filters”, associated with a perspective that these compounds function by UV-light absorption, while the term “physical filter” has sometimes been used to describe mineral nano and microparticles, associated with a protection mechanism against UV light via scattering. This is inaccurate because inorganic mineral filters have also been demonstrated to have absorption as an important mechanism of protection [13]. In addition, some organic filters, such as bisoctrizole, function by both scattering and absorption mechanisms. Also, the term “chemical filters” might imply that mineral filters are not chemicals, which is of course inaccurate. Therefore, we will be using terms “small-molecule” and “mineral” to differentiate filter type when needed.

Public perception of sunscreen safety has driven the market to use certain ingredients in abundance while limiting others, based upon relatively little data. Two trends have emerged in recent years because of public perception. First, oxybenzone has been essentially discontinued due to concern over its hazards to coral reefs [2]. On January 1st, 2021, the State of Hawaii prohibited the sale and distribution of sunscreen formulations that contain either oxybenzone or octinoxate [14]. Second, inorganic sunscreens containing TiO_2_ and ZnO are increasingly marketed as safer alternatives to small-molecule (“chemical”) sunscreens [5, 6]. However, claims of product safety appear to ignore the hazards that can result from UV irradiation of metal oxides in these products, including well-documented generation of reactive oxygen species (ROS) [15, 16] and degradation of organic compounds [17].

In contrast to the United States (US), the European Union (EU) has 28 approved UV-filters for inclusion in cosmetic products: nine UV-B-absorbing organic compounds; seven organic compounds that absorb UV-B and short-wave UV-A; four UV-A-absorbing organic compounds; and four organic compounds that provide broad-spectrum UV absorbance. The use of both TiO_2_ and ZnO is also approved, including their use as nanoparticles (with certain hazard labels, specifications and concentration restrictions) [4]; however, TiO_2_ was also recently classified under EU Regulation as a category 2 suspected carcinogen by inhalation [7], with warnings now required on associated products containing inhalable TiO_2_. It is not clear how spray-on sunscreens fit within this modified regulation, nor how long TiO_2_ will remain approved for use in any sunscreen application. In addition, the EU allows the use of two organic compounds, bisoctrizole and *tris*-biphenyl triazine, that are formulated in nanoparticulate form, enabling them to function as physical–chemical hybrids, providing both UV absorbance and scattering [18, 19]. The larger array of approved compounds available to formulators in the EU motivated us to study the safety of both US and EU ingredients, with the goal of determining strategies for minimizing formula hazard.

While consumers have become aware of the potential hazards of sunscreen ingredients, an area of importance that has yet to receive public attention is the photodegradation of sunscreens/UV-filters and the toxicity of the degradation products. UV-filters have been observed to undergo UV-induced chemical degradation; however, the timeframe and extent of these reactions is dependent upon each formula’s composition [20–24]. The most common UV-filters have undergone photostability testing and formulation strategies have been developed to prevent their rapid decomposition [20]. UV-A filters, in particular, are generally not photostable and rapidly degrade upon UV exposure, resulting in a marked reduction in UV-absorbance, and thus their efficacy [25, 26]. For example, avobenzone, one of the few FDA approved UV-A filters, is known to undergo photodegradation [25]. The addition of octocrylene, a UV-B and short-wave UV-A filter, can help stabilize avobenzone when a sufficient amount is added [27, 28]. The effect that photodegradation products have on formula toxicity is not well-understood [20]. When a sunscreen ingredient is determined to be non-toxic and safe for formulations, the assessment is only based on an evaluation of the pure chemical, and not any photochemically generated species. Considering that there are a number of studies demonstrating that sunscreens can quickly react under UV-exposure [20, 24, 27–30], the specifically intended setting for use (for example, outside on a sunny day), it is surprising that very little toxicity testing has been done on the photodegradation products [20, 29].

Herein our aim was to study the photodegradation, and toxicity following irradiation, of commercially relevant UV-filter mixtures from both the EU and US. Spectroscopic analysis elucidated how mixture composition and UV protection were affected by UV light. The toxicities of the mixtures were evaluated in embryonic zebrafish assays. The choice of zebrafish as a model organism was based upon the significant gene homology to humans and the ability to conduct higher throughput screening compared to mammalian studies [31]. More rapid screening allowed us to perform an in vivo study that tested a range of mixture combinations in a statistically significant manner. Additionally, zebrafish models are routinely employed for understanding aquatic ecotoxicology [32]; therefore, zebrafish assays can be used to predict the hazards posed by sunscreen degradants in aquatic ecosystems.

## Materials and methods

### Materials

All chemicals purchased were of cosmetic-grade or above. International Nomenclature of Cosmetic Ingredients (INCI) ingredient names are included, when different from common names. Homosalate (CAS: 118-56-9), octocrylene (INCI: 2-ethylhexyl ester; CAS: 6197-30-4), oxybenzone (INCI: Benzophenone-3; CAS: 131-57-7), and avobenzone (INCI: Butyl methoxy dibenzoylmethane; CAS: 70356-09-1) were purchased from makingcosmetics.com. MakingCosmetics Inc. is a FDA-registered, ISO certified, and OTC-licensed cGMP/FDA ingredient supplier, based in the United States that supplies businesses and individuals with cosmetic ingredients [33]. Octisalate (INCI: Octyl salicylate; CAS: 118-60-5) was purchased from TCI Chemicals. DHHB (INCI: Diethylamino hydroxybenzoyl hexyl benzoate; CAS: 302776-68-7; received as UVINUL^®^ A PLUS) and Bisoctrizole (INCI: Methylene bis-benzotriazolyl tetramethylbutylphenol; CAS: 103597-45-1; received as TINOSORB^®^ M, a 50% aqueous suspension of the UV-filter) samples were acquired from BASF. The microparticulate ZnO (referred to as ZnO microparticles herein; CAS: 1314-13-2) was purchased from makingcosmetics.com; it is described as free of other metal impurities, with particle sizes ranging 200–1000 nm, and prepared by a high-temperature vaporization of zinc. It is recommended to be added to products in 5–25% *w*/*w* concentrations, with a maximum US limit of 25%. The nanoparticulate ZnO (referred to as ZnO nanoparticles herein; CAS: 1314-13-2), purchased from makingcosmetics.com, has a commercial name of “micronized ZnO” but is described as having particles < 100 nm in size, with a mean size of 85 nm; it is recommended to be added in 3–6% *w*/*w* to organic sunscreens or 3–20% *w*/*w* when used alone. Both types of ZnO particles were purchased in a form described as “uncoated”, which is a different from “coated” forms where the ZnO is coated with a silicone derivative.

### UV-filter mixture formulation

We evaluated the ingredients of 26 commercial sunscreens from both the US and EU markets. From these data, we identified trends in commonly combined UV-filters (active ingredients) and designed five commercially relevant mixtures of UV-filters (Table 1). We determined the concentration of each filter by using BASF’s online sunscreen simulation tool [34] to generate formulas that were predicted to have a sun protection factor (SPF) of 15 (± 0.4). The BASF tool also applies “Pass/Fail” criterium based on the EU, AUS, and MERCOSUR protocol that compares the UVA-protection factor in vitro (ISO 24443) and UVA-protection factor in vivo (ISO 24442) and considers the higher value [34] to determine whether the formulation will achieve a suitable level of UV-A protection. All of the mixtures were designed to receive a UV-A “Pass” by this criterium.

**Table 1:** UV-filter mixtures formulated to achieve a calculated SPF of 15

	*% w/w* of UV-filter^a^ in mixture						
Mixture number	Avobenzone	Octisalate	Homosalate	Octocrylene	Oxybenzone	DHHB	Bisoctrizole
1	1.8	4.0	7.0	5.0	0.0	0.0	0.0
2	1.0	4.0	0.0	3.0	0.0	0.0	3.0
3	2.0	3.0	3.0	4.0	0.0	5.0	0.0
4	2.0	3.0	6.0	3.0	2.5	0.0	0.0
5	0.0	3.0	0.0	2.0	0.0	0.0	5.0

^a^INCI names are included in Sect. 2.1

To formulate mixtures the raw chemicals were weighed and solvated into neat dimethyl sulfoxide (DMSO). DMSO was selected as a solvent because these mixtures were eventually going to be assessed in zebrafish toxicity assays, and DMSO is a common and well-tolerated co-solvent for delivering water insoluble chemicals to zebrafish [35]. While DMSO would never be found in commercial sunscreens, it ensured effective delivery of the chemicals to the fish, is non-toxic to zebrafish at the quantity used in the assays, and was included in background controls to ensure any minor effects of the DMSO were accounted for in the data. The UV-filter solutions were combined with one another and an appropriate amount of DMSO was added to bring the final concentrations of chemicals to the amounts stated in Table 1, with a total of 3 g of each mixture.

To formulate the ZnO-containing mixtures, small aliquots of the 3 g “mixture 1” stock were combined with 6% (*w*/*w*) of ZnO particles (microparticles or nanoparticles). The addition of ZnO resulted in thick suspensions, so suspensions were vortexed immediately prior to pipetting to ensure representative sampling of the mixture. The ZnO-containing mixtures were irradiated and diluted using the same procedure as for the small-molecule mixtures.

The lotion for the spectrum in Figure S1 was formulated according to a typical industry method for formulating sunscreen lotions [36], with each component described below in percentage terms representing the relative mass of each ingredient by weight (*w/w*) in the final formulation (total 100%). An aqueous phase was prepared by dissolving glycerin (3%) and disodium EDTA (0.2%) in water (65.7%) with stirring, and heated to 75 °C. Separately, an oil phase was prepared by mixing C12-15 alkyl benzoate (8%), cetyl alcohol (2%), xanthan gum (0.3%), glyceryl stearate (1%), ceteareth-20 (2%), avobenzone (1.8%), homosalate (7%), octisalate (4%), and octocrylene (5%) with stirring, and heated to 75 °C until all solids were dissolved. The two phases were then combined at 75 °C by mixing with a homogenizer until an emulsion was achieved. The resulting emulsion was then cooled to room temperature (~ 25 °C) with stirring [36]. It is worth noting that any degradation of lotion ingredients that may have been caused by heating would be accounted for experimentally because all spectra (both before and after UV exposure) were collected for lotions post-formulation and cooling (Figure S1).

### UV-exposure

All mixtures were freshly vortexed before use to promote homogeneity, then 3 µL aliquots were removed and placed into small glass vials without any tapering to ensure the UV-beam would not be obstructed by the vial. The vials were exposed to a solar simulator (Newport Oriel Sol3A) using the AM1.5 G incident spectrum.^1^ The vials were opened and exposed to the solar simulator, with a measured total power density of 104 mW cm^−2^, for 120 min. Using the standard 1.5 G solar spectrum data available on The National Renewable Energy Laboratory’s website [37] and the erythema spectral weighting function reported in ISO/CIE 17166 [38, 39], it was determined that 0.018% (equivalent to 0.019 mW cm^−2^) of this solar-simulated spectrum is erythemally effective radiation. By multiplying this effective power density by exposure time (7200 s), the value can be converted to a Standard Erythemal Dose (SED). The SED is defined as 100 J m^−2^ of erythemally relevant exposure [40], therefore, 13.8 SEDs were delivered by the solar simulator to the samples over a two hour period. As the exposure level is directly relevant to human UV exposure, it is important to contextualize this in relation to UV Index (UVI).^2^ A simple mathematical relationship exists between UVI and the number of SEDs: hourly erythemal dose (J m^−2^) = 90 × UVI (J m^−2^) = 0.9 × UVI (SED) [41]. Thus, if the UVI is 10, an individual in full sunlight will receive 9 SEDs per hour; if the UVI is 6, ∼ 1 SED is delivered every 11 min. Therefore, the 13.8 SEDs delivered by the solar simulator in our experiments herein are equivalent to 92 min of exposure at UVI 10, or 153 min of exposure at UVI 6.

Control experiments showed that after 2 h of exposure there was no significant evaporation of DMSO. Following exposure of test samples, 97 µL of DMSO was added to the vials and vortexed. These solutions were then used for toxicity and spectroscopic analysis. Control samples, not exposed to UV irradiation, were prepared in an identical manner except they were kept open in the dark during the irradiation period, which was done because of the hygroscopic nature of DMSO to account for any water absorbed over the two-hour period.

### Absorbance measurements

Aliquots of irradiated and control mixtures were removed from the 100 µL vials and diluted into 99% water or isopropyl alcohol (IPA). 200 µL of the 99:1 solutions were placed into UV-STAR^®^ microplates for measuring the absorbance. A BioTek Synergy 2 microplate reader was used with Gen5 1.11 software. Scans were run between 280 and 700 nm in 2 nm steps, and the 99:1 solvents were background subtracted. Only the UV region (280–400 nm) is displayed within the included spectra because longer wavelengths had no absorbance, even following degradation. The absorbance spectra had a 3% variation when performed in triplicate.

### Preparation of solutions for animal exposure

Glass vials containing 50 µL of each concentrated mixture in DMSO were placed in 50 mL falcon tubes and centrifuged at 64×*g* for 3 min. Following centrifugation, the bottom of the tubes were lightly flicked on the outside using a pointer finger to help mix the chemicals. To achieve 10 × exposure solutions, the samples were diluted into a mixture of ultrapure (UP) water and DMSO to achieve the desired concentrations. 10 µL of each 10 × exposure solution was then added to 90 µL of UP water in each individual well to reach a final concentration of 1% DMSO and the mixture concentrations listed in Table S1.

### Zebrafish husbandry/developmental exposures

Tropical 5D wild type zebrafish were housed at the Sinnhuber Aquatic Research Laboratory (Corvallis, OR) at Oregon State University under a 14 h light/10 h dark cycle. Fish were raised in tanks with ~ 500 fish/50-gal tank filled with reverse osmosis water supplemented with Instant Ocean (0.6%) and kept at 28 °C (± 1 °C). Their diet consisted of appropriately-sized Gemma Micro (Skretting Inc, Tooele, France) fed to them two times a day. Zebrafish were group spawned in tanks with spawning funnels placed in the tanks the night before, and embryos collected the next morning. The embryos were staged according to a previously described procedure [42] and kept in an incubator at 28 °C in embryo media. Embryo media’s composition was 15 mM NaCl, 0.5 mM KCl, 1 mM MgSO_4_, 0.15 mM KH_2_PO_4_, 0.05 mM Na_2_HPO_4_ and 0.7 mM NaHCO_3_ [43]. At 4 h post-fertilization (hpf), the chorions were removed with the use of an automated dechorionator and 83 µL of 25.3 U µL^−1^ of pronase (Roche, Indianapolis, IN, USA) [44]. The embryos were transferred to individual wells of 96-well plates containing 100 µL of the exposure solution where they were statically exposed until 120 hpf (*N* = 12). The plates were sealed with parafilm and shaken overnight at 235 rpm. The embryos were assessed for a total of 22 endpoints at 24 and 120 hpf [45].

## Results and discussion

### UV-filter mixtures containing small-molecules

**Chart 1:**
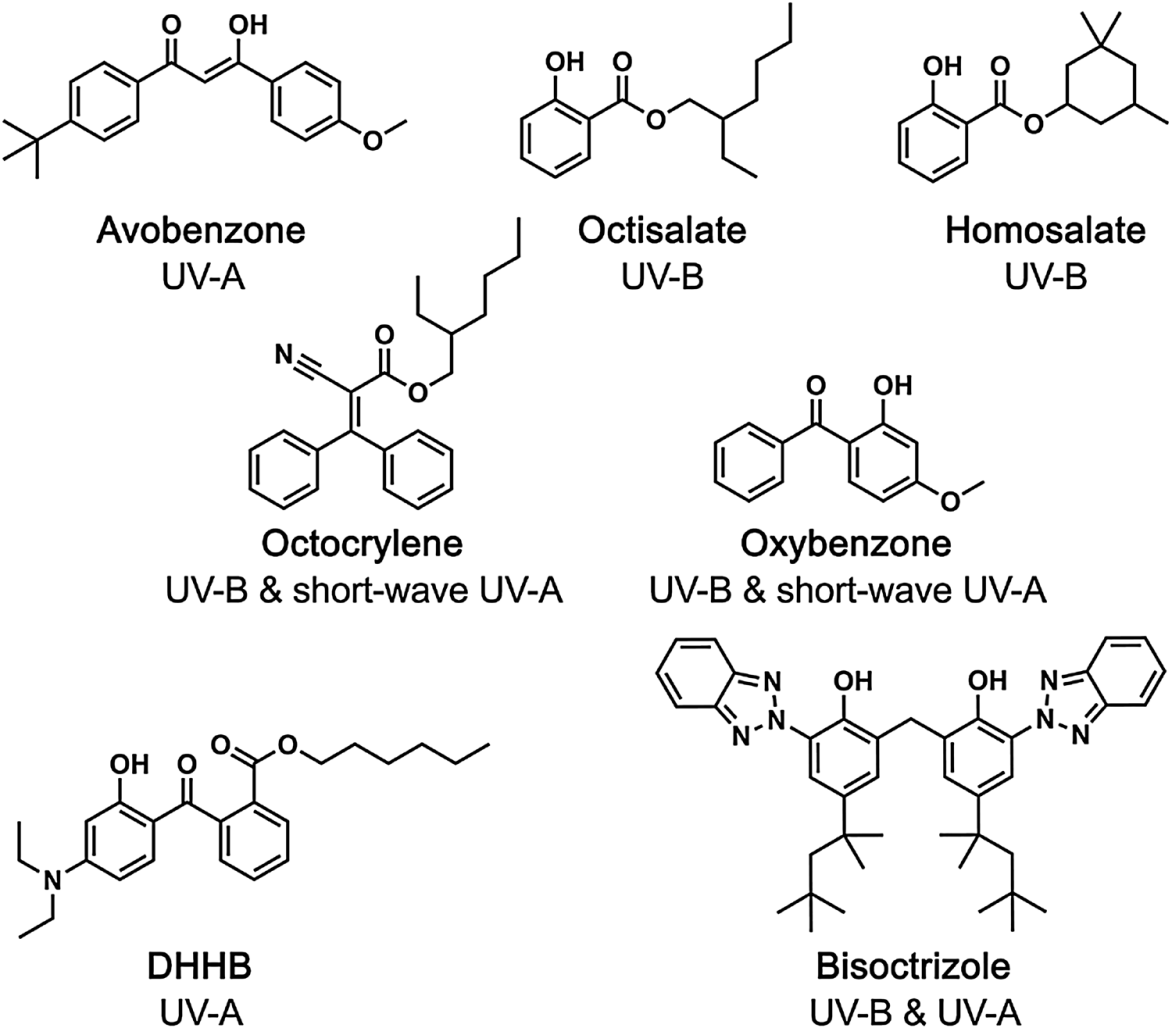
Structures of the UV-filters used in the small-molecule formulations and the type of UV light they absorb

Five different small-molecule based UV-filter mixtures were formulated to have an SPF of 15 (formulations are detailed in Table 1, UV-filter molecular structures are shown in Chart 1). These mixtures were designed based on a significant market review of commercially available products in the US and EU; using the ingredient information on the bottles, we identified trends in the most common formulation types, and then formulated our own mixtures based on typical ingredient compositions. Mixture 1 represents a formulation used commercially for “sport” applications and is very commonly found on the market in both EU and US. Mixtures 2 and 3 represent a “sport” lotion that also incorporates UV-filters approved in the EU but not the US (bisoctrizole and DHHB). Mixture 4 represents typical sunscreen products found on the market in both US and EU that combine many UV-filters (usually to achieve a high SPF, but here their concentrations were intentionally low to normalize SPFs between mixtures). Mixture 5 represents a formulation for allergy-sensitive skin using a filter available in the EU, but not US.

All mixtures were formulated in DMSO, as described in Sect. 2.2. It is worth noting that past studies have found that the extent of UV-filter photodegradation is solvent dependent, with polar solvents generally reported as more stabilizing than non-polar solvents, therefore even more degradation might have resulted had we used a non-polar solvent [20]. Despite DMSO’s polarity (and stabilizing effect), the stability of the mixture depends upon the formulation because identity-dependent UV-filter interactions with one another can promote or hinder photodegradation [46]. In the range of ~ 7–18% active ingredients, and for 120 min of UV irradiation, the mixtures were stable when the proportion of octocrylene was high enough to protect the avobenzone from photodegradation. Past work has found that octocrylene likely hinders avobenzone photodegradation by quenching the excited triplet state of the avobenzone β-diketo isomer (formed upon irradiation); but, other stabilizing processes can also occur [46]. We did observe photodegradation when the ratio of avobenzone: octocrylene was larger than in mixture 1 (see Figure S2).

**Fig. 1:**
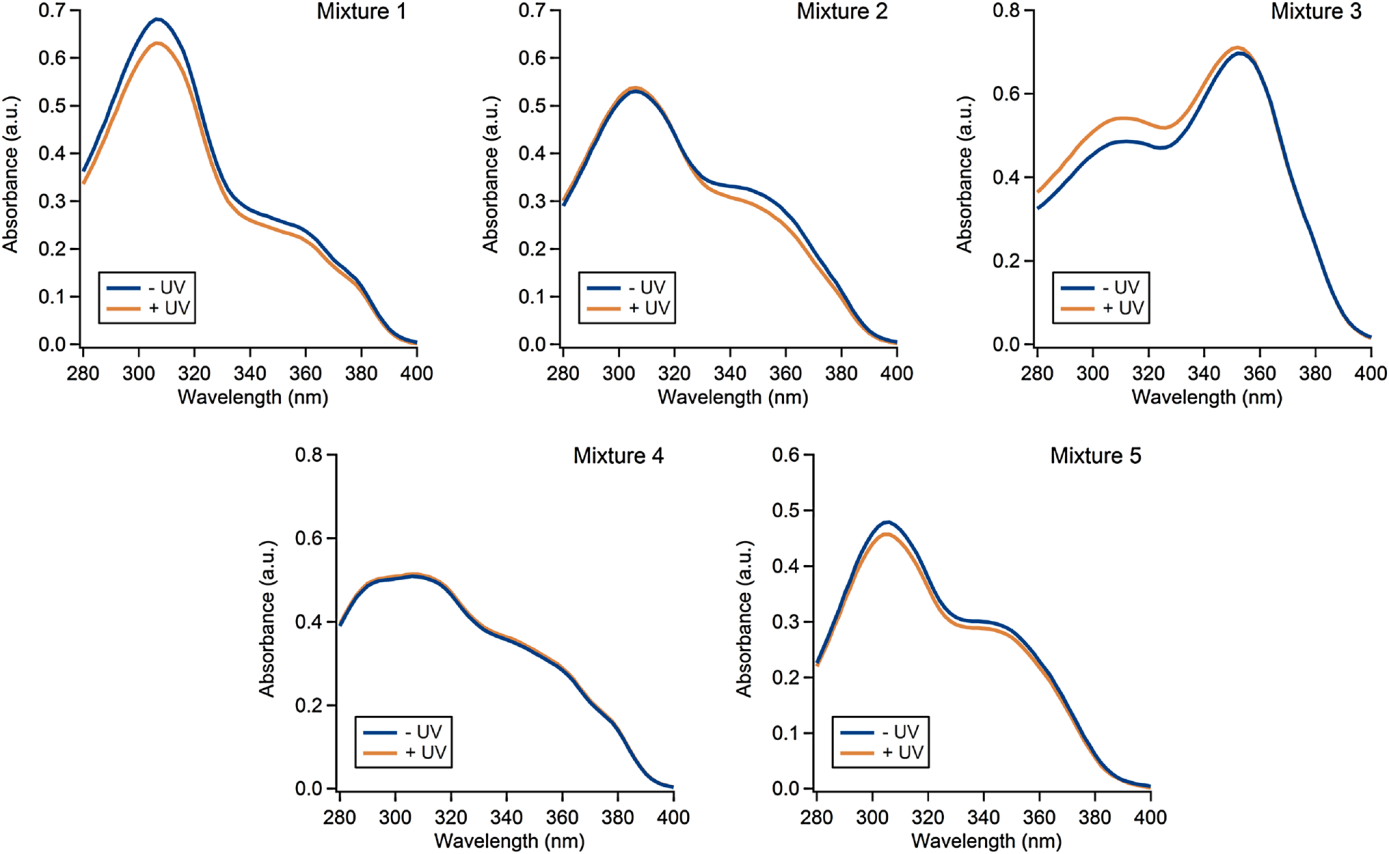
UV–Vis spectra showing photodegradation of mixtures 1–5 measured in 99:1 IPA/DMSO

Mixtures 1–5 were irradiated with a solar simulator using conditions representative of a clear sunny day, as described in detail in Sect. 2.3. The irradiated mixtures, and non-irradiated controls, were diluted in DMSO then mixed with 99 parts of IPA or UP water. UV–Vis spectra were collected in both 99:1 IPA/DMSO (Fig. 1) and 99:1 water/DMSO (Figures S3–S4). In the water/DMSO samples we observed a high baseline trace, characteristic of scattering, suggesting that this solvent system did not fully solubilize all the chemicals. Thus, the photodegradation was examined in two solvent systems that each provided different information. IPA solubilized all of the mixture components, thereby giving a complete picture of the chemical degradation, while the 99:1 water/DMSO system showed the chemical exposure that zebrafish embryos experienced during the toxicity assays. Overall, the results from the two solvent systems suggest that the mixtures were mostly photostable, however, the results in IPA/DMSO were more straightforward to interpret because the solutions were homogeneous.

UV–Vis absorbance spectra are informative for assessing UV-filters because they not only provide data on ingredient degradation but also on product performance. The efficacy of a small-molecule based sunscreen can be determined by its UV–Vis absorbance. A sunscreen product should have good absorbance throughout the entire UV-A and UV-B regions (280–400 nm) and if the mixture is photostable then the absorbance spectrum should not decrease or change shape after exposure to UV irradiation.

The UV–Vis data collected on mixtures 1–5 show that these mixtures exhibit little photodegradation despite containing avobenzone, which is known to undergo photolysis individually [20, 30]. These findings suggest that the small-molecule based formulas available commercially, which were the basis for the formulas studied here, are formulated with ratios of ingredients that minimize photodegradation. The presence of octocrylene in each of the five mixtures may have had a significant stabilizing effect, thus hindering photolysis [28]. We did not initially anticipate octocrylene would be this influential because past work has shown that even when adequately stabilized, a modest amount of photolysis still occurs (e.g. ~ 16% for avobenzone in the presence of octocrylene) [20]. However, it is difficult to draw direct comparisons when all other references use highly varied irradiation conditions and solvents.

The individual UV-filters were screened through in vivo zebrafish assays at various concentrations to determine appropriate doses for eliciting an effect on animal development. Based upon these studies, we selected doses between 0.00142% and 0.003% (*weight of UV-filters/weight of solution*) depending on the mixture. Irradiated mixtures were always tested at the same concentration as their non-irradiated control.

**Fig. 2:**
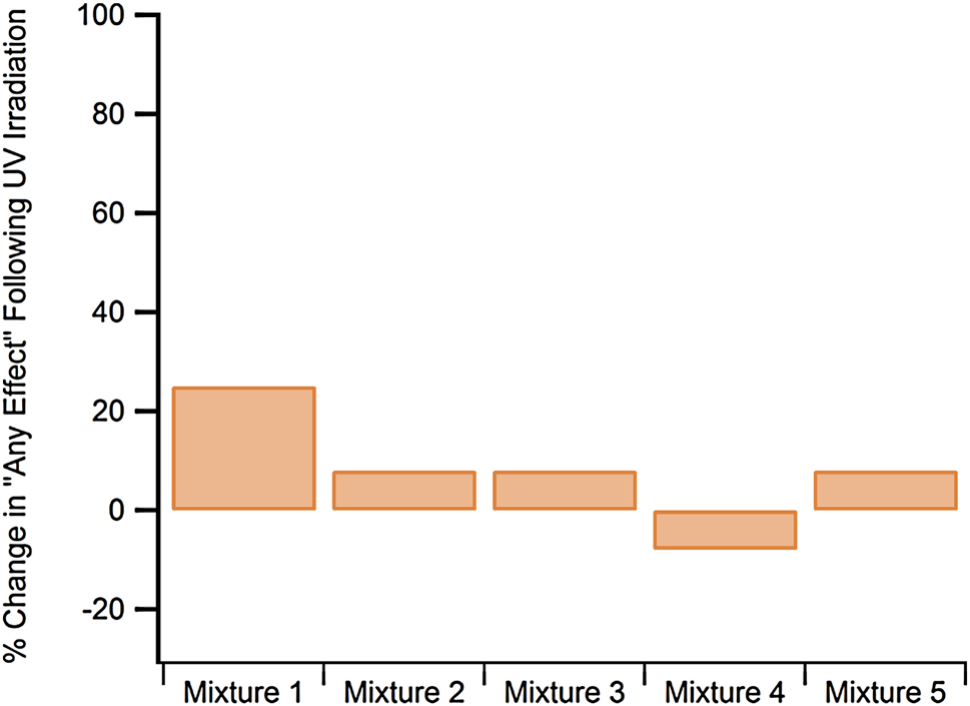
Summary of changes in toxicity to mixtures 1–5 following UV irradiation

The zebrafish were exposed to each mixture for 5 days and 22 developmental endpoints were monitored. Because photodegradation, and consequent degradant toxicity, was insignificant for these mixtures, the developmental results have been aggregated into a single endpoint in Fig. 2. The *y*-axis represents the difference in toxicity between the irradiated and non-irradiated mixtures. In this case, the “toxicity” is a single endpoint that is the summation of all morphological and mortality effects experienced by the fish. We aggregated the data because the differences were so low that looking at one morphological or mortality endpoint was not informative.

The toxicity data are in good agreement with the spectroscopic data. UV irradiation of mixtures 1–5, which do not contain any mineral UV-filters, elicits minimal differences in formulation UV absorption properties, as shown by the spectra in Fig. 1, and toxicity, as shown by the data in Fig. 2. Following irradiation, only mixture 1 has a change in toxicity that is > 10%. The differences in toxicity for mixtures 2–5 are all minor at ± 8%. Even for mixture 1, the 25% difference in toxicity is minimal considering this is an aggregated endpoint.

It is important to note that the set of experiments just described were conducted on mixtures of chemical UV-filters without the non-active ingredients found in lotions such as emollients, surfactants, and preservatives. The experiment was designed this way to focus on the nature of the UV-filters. We initially set out to formulate complementary lotions as well, but preliminary results suggested that obtaining reliable photodegradation data from the lotions would not be possible within the scope of this work. The challenge with formulating lotions is that their degradation is highly dependent upon film thickness. We formulated a generic body lotion base (described in Sect. 2.2) and added the organic actives to the oil phase prior to heating [36]. The lotion was spread, using a clean nitrile-gloved finger on a glass microscope slide, into a film of 1.5 mg cm^−2^ thickness, which falls at the high end of average consumer use but below the 2 mg cm^−2^ standard for determining SPF [47]. The film was exposed to UV irradiation for 2 h before being solvated in DMSO and diluted in water to measure the UV–Vis spectrum. Using the lotion method, it was clear that sun protecting ability was diminished upon UV irradiation, but the absorbance values that resulted from the method (Figure S1, right) were not within an appropriate analytical range and outside of Beer’s law (*A* at *λ*_max_ < 0.05); in addition, the irradiated lotion films did not yield a sufficient sample quantity for a full toxicity assay, and spreading the films uniformly was a challenge. A reduction in sun protecting ability was also observed with the neat actives in DMSO, without lotion components (Figure S1, left). Therefore, it was decided that the degradation of the UV-filters in DMSO, without the lotion components, was a reasonable way to measure the photodegradation and would be where we focused our efforts for this study.

### UV-filter mixtures containing small-molecules and ZnO

The performance of a small-molecule based sunscreen could be influenced by the presence of ZnO particles either via intentional mixing in hybrid sunscreens (containing both small-molecule and mineral UV-filters) or through incidental mixing when cosmetics and/or different sunscreens are used in combination. While metal oxide particles have been documented to generate ROS and induce small-molecule degradation [15–17], little attention has been paid to how this may affect sunscreen toxicity. To investigate this, one small-molecule based UV-filter mixture was examined with two different sizes of ZnO particles added. Of the five small-molecule-based mixtures examined in this work, mixture 1 was the most representative of current commercial formulations, with relevance in both the US and EU markets, so we used this mixture as the model for studies with ZnO particles.

**Fig. 3:**
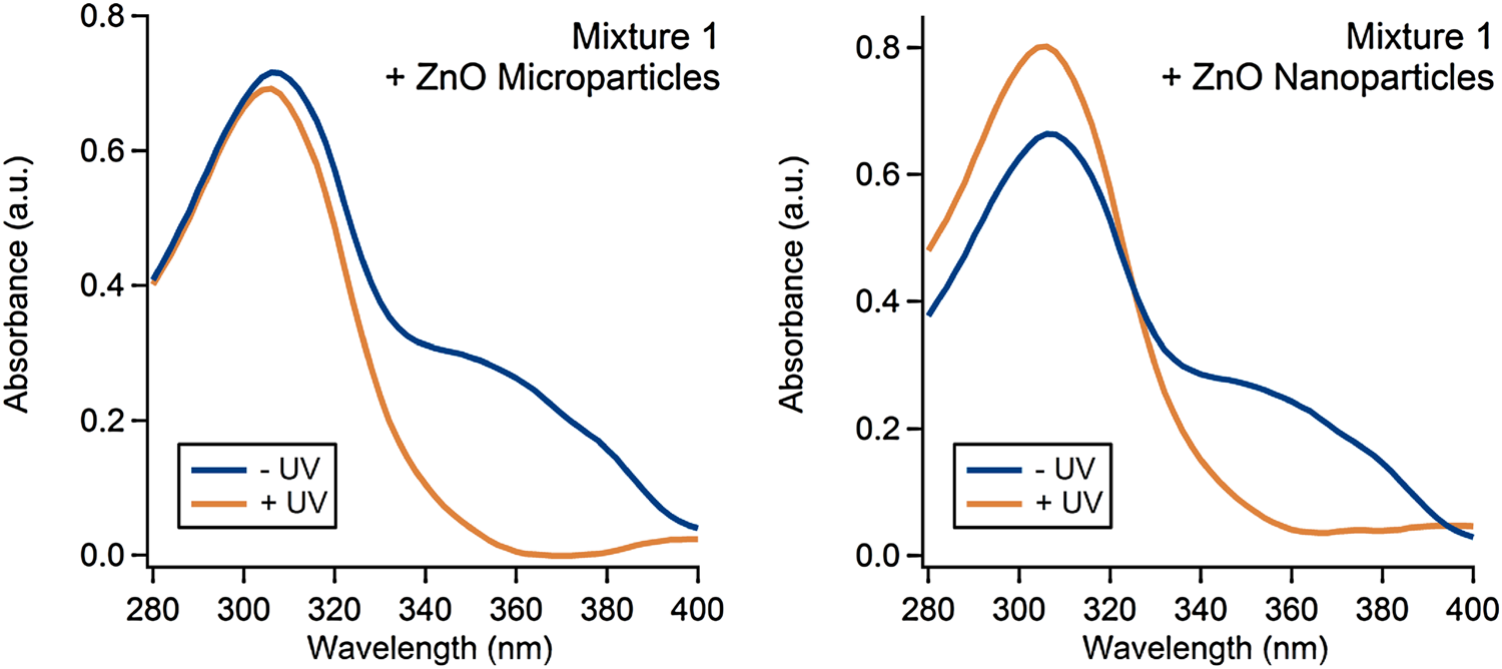
UV–Vis spectra of mixture 1 + ZnO particles before and after 2 h of UV irradiation

Two different sizes of ZnO particles were examined with mixture 1: microparticles with sizes ranging 200–1000 nm and nanoparticles with sizes < 100 nm. Both sets of ZnO were reported to be prepared via a high-temp vaporization synthesis and neither were coated. The particles were added to mixture 1 in 6% (*w/w*) quantities, which is a typical amount for a hybrid sunscreen. Following particle addition, the mixtures were exposed for 120 min of UV irradiation and then their UV–Vis spectra were measured (Fig. 3). Since the particles do not form a homogenous solution with either IPA or water, minor differences in the baseline can be attributed to the solution heterogeneity imparted by the particles.

**Fig. 4:**
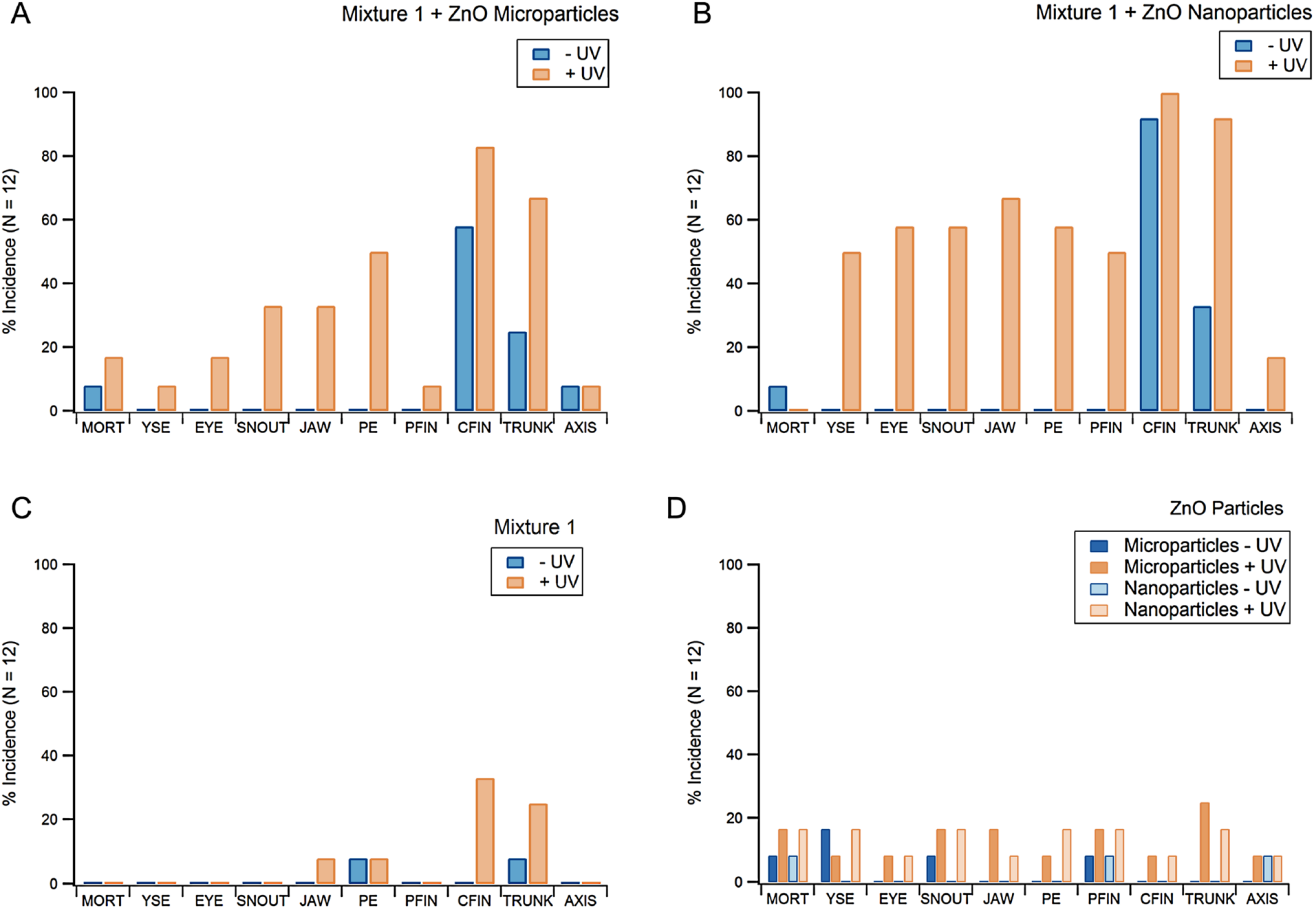
Changes in zebrafish development over five days at ten different endpoints. Animals were exposed to 99:1 water/DMSO solutions containing 0.0014% (*w/w*) organic filters (mixture 1; panels **a**–**c** and 0.0005% (*w/w*) ZnO (panels **a**, **b** and **d**). Key to endpoints: mortality (MORT), excess fluid accumulation around yolk sac (YSE), abnormal eye placement or size (EYE), visibly malformed snout (SNOUT), jaw (JAW), excessive fluid accumulation around pericardial edema (PE), under developed or malformed pectoral fin (PFIN) or caudal fin (CFIN), body length shorter than normal (TRUNK), and body axis curvature (AXIS)

Following UV irradiation the lower energy absorbance peak (350–400 nm) disappears; this is consistent with avobenzone degradation [17, 20]. Avobenzone was the only longwave UV-A absorbing small-molecule present in mixture 1 so there was clearly a change in avobenzone’s structure that resulted in the mixture’s loss in UV-A absorbance. Avobenzone is known to undergo keto-enol tautomerization, wherein its enol-form (UV-A absorber) converts to the diketo-form (UV-C absorber) and then can undergo various lysis reactions [20, 27]. It is possible that the spectral change could be due to avobenzone tautomerization rather than any molecular cleavage, but taking the spectroscopic data and toxicity data (Fig. 4) together suggests that is not the case. Since we did not observe major changes in the photostability until the ZnO was added, it is likely that the UV irradiation produced electron–hole pairs in the ZnO, leading to the generation of ROS [16] and subsequent oxidative degradation of avobenzone. Additional mixture 1 UV-filters that absorb 280–350 nm light may have also been degraded by ROS, but their overlapping spectroscopic signatures preclude the ability to draw any conclusions from these data.

The UV-A protection factor (UVAPF) was calculated using ISO 24443:2012, an in vitro method for determining sunscreen UV-A protection [48], using measured absorbance values between 320 and 400 nm and corrected to account for the pathlength and concentrations used in the microplate assays. The percent change in UVAPF due to UV exposure was determined. For mixture 1, the addition of ZnO microparticles caused a 91.8% loss in UVAPF; whereas, the ZnO nanoparticles caused an 84.3% loss. This is in stark contrast to mixture 1 by itself, that only showed a 15.8% loss in UVAPF. Mixture 1 + ZnO contained just 6% ZnO, which is a lower concentration relative to sunscreens on the market that contain ZnO as the only UV blocker (typically 12–24%). Therefore, the ZnO particles in our hybrid mixtures are not expected to safely protect skin against UV-A damage once the organic filters in mixture 1 have been degraded. A summary of the results and a description of the calculation can be found in the supporting information.

Besides significantly diminishing UV-A protection, exposing ZnO + mixture 1 to sunlight also increased hazards by producing toxic photodegradation products (Fig. 4). The experimental design and controls make it clear that this toxicity is not the result of the pure inorganic particles or UV light. Any ROS generated during UV irradiation would not have been present during animal exposure because the mixtures were exposed to UV light days prior to the zebrafish assay. Instead, we can definitively state that ZnO induces the production of toxic photodegradants. This is supported by the control experiments that show only minor amounts of toxicity observed for the organic and ZnO components alone (Fig. 4 C and D). Panel C in Fig. 4 suggests there is a slight increase in mixture 1 toxicity following UV irradiation, as was discussed in Sect. 3.1. Panel D shows that the UV light may have induced a small amount of damage to the ZnO particles that made them more toxic; this could happen by etching them and/or leaching toxic Zn ions [49]. The results from panels A and B in Fig. 4 are not additive from panels C and D though, there is clearly an increase in toxicity due to photodegradation of small-molecules that is induced by ZnO. This is further suggested by the significant difference between the orange and blue trace in each spectrum in Fig. 3. The 22 endpoints monitored in vivo provide a comprehensive method of identifying developmental effects in an integrated system. The morphological malformations observed provide insight into the biological targets of these mixtures. Studying these targets is useful for guiding any future investigations into the mechanism(s) of toxicity. Because we observed a high incidence of morphological effects but not mortality, the mechanism(s) of toxicity is likely linked to changes in biological signaling systems.

## Conclusions

The aim of this study was to establish if certain sunscreen ingredients or formulations undergo photodegradation that can be harmful to humans and/or the environment. We were surprised to find that all five of the commercially relevant small-molecule UV-filter mixtures were mostly photostable. These results suggest that the ability of the small-molecule formulas to protect against UV-damage is not altered under normal use conditions. This may be because the ratios of actives have been industrially optimized to minimize photodegradation, although such findings have not been reported in the literature. This small-molecule mixture stability was further observed during in vivo analysis, which indicated there were minimal differences in biological impacts following UV irradiation. However, when a widely used small-molecule formulation was studied in combination with a modest amount of ZnO particles (6%; commercial recommendations are 5–24%), significant differences in photostability and phototoxicity were observed. Both the nanoparticulate and the microparticulate ZnO degraded the organic mixture and caused > 80% loss in organic filter UV-A protection. In addition, the ZnO-induced photodegradation products caused significant increases in zebrafish morphological defects. These results suggest that ZnO particles may increase sunscreen toxicity in ways not currently recognized.

The results show that formulas containing both ZnO and small-molecule UV-filters can undergo photochemistry that results in two different types of deleterious effects; they can have significantly decreased UV-A protection due to degradation of the organic UV-filters, and they can generate toxicity-inducing photodegradation products. Loss of UV-A protection is especially problematic in US sunscreens where the list of approved filters is so small; only avobenzone and zinc oxide are regularly used for long-wave UV-A protection in commercial US sunscreens [50, 51]. This finding motivates the need to identify additional UV-A absorbers that can be approved for use in the US. Some of the European ingredients such as bisoctrizole and DHHB show promising photostability [4, 52] and have no reported toxic photodegradation products, currently. Hopefully, the pipeline of US-approved UV-filters can be strengthened through continued study of promising chemicals. Even in a best-case scenario where new UV-filters end up being better performing and safer than current options, obtaining regulatory approval is time-consuming and expensive [51]. In the meantime, further work should be done exploring the phototoxicity of various architectures of coated ZnO. Prior work has indicated that the photocatalytic activity of titanium dioxide particles can be minimized by coating them with silica or aluminium hydroxide [20], so similar strategies may be helpful for minimizing ZnO-induced photodegradation.

Both nanosized ZnO particles and non-nano ZnO particles caused toxicity upon UV irradiation. As a team that specializes in studying nanoparticle toxicity, these results are not surprising to us. We suspect though, that these results would surprise many consumers who are misled by “nano free” labels on mineral-based sunscreens. Our findings suggest that any size metal oxide particle can have reactive surface sites, whether it is less than 100 nm (generally determined to be “nano” sized) or not. More important than metal particle size, is the metal identity, crystal structure, and any surface coatings [15]. We acknowledge that including these properties on a product label is not practical; however, it is inappropriate to mislead the public about safety through marketing tactics focused on particle size. This is especially relevant now that the EU has recently classified TiO_2_ nanoparticles as a suspected carcinogen by inhalation [7], a change that is likely to push the market towards substituting TiO_2_ with larger TiO_2_ particles or ZnO in cosmetics. Considering the results of this work, and the likelihood of nano-sized ZnO also being an inhalation hazard, formulators should be cautious about rapid, widespread formula modifications unless there is evidence that specific metal oxide sizes, compositions or architectures offer improvements in product performance and safety.

In our testing, we considered a comparison with sunscreen lotions bought over the counter that contained both ZnO and small-molecule-based UV-filters. However, commercial products have limited information available on the levels used of each ingredient, particularly in the EU, and the inclusion of preservatives, fragrance, and other additives would have convoluted the results. Furthermore, the size of any mineral particles, and any potential coatings, is not specified in these commercial products. Finally, the formulation date and any conditions that the bottle may have been exposed to could influence formula stability, thus expanding the number of samples necessary for robust testing. For these reasons, we decided there would be too many parameters to meaningfully interpret results of a commercial product and make either quantitative or qualitative comparisons to the mixtures studied herein. Further work on the UV stability of commercial formulations would be useful, but only where full details of commercial formulations are available.

This study found that combining sunscreen active ingredients, which are safe on their own, can result in decreased mixture safety following UV irradiation. With the global sunscreen market forecasted to reach 24.4 billion dollars by 2029 [53], and consumers paying attention to formula ingredients [6], it is important that scientific data guides sunscreen design and not unfounded consumer demand. While consumer concerns have led to some positive improvements, such as broad-spectrum protection labeling [54], they have also enabled misleading marketing like the promotion of “chemical-free” sunscreens. Moreover, SPF labels are included in more than just sunscreen lotion; they are now regularly found on an array of cosmetic products that are intended for daily use in combination such as, facial moisturizer, liquid foundation and powder foundation. Currently, there is no awareness that mixing products may increase product toxicity. We fear that the increasing ubiquity of UV-filters (in particular metal oxide particles), coupled with the lack of studies on sunscreen phototoxicity, especially as formulated products, is likely to result in products that have unintended consequences and regrettable chemical substitutions [55]. We observed that photodegradation products resulted in increased mixture toxicity to zebrafish, thereby suggesting that degradant introduction into aquatic ecosystems may be environmentally hazardous. Hopefully, this work can bring awareness to some of the hazards of UV-filters and caution against their widespread incorporation into products where sun protection is not crucial, especially until there is more information on how to design sunscreens for degradation that does not result in an increase in ingredient toxicity.

Overall, much more work studying sunscreen formula photostability and phototoxicity is needed to guide design and mass production of safe and effective formulations. Such stability and toxicity studies should inform any sunscreen reformulations, or changes in UV-filter policy, so that regrettable chemical substitutions are avoided.

## Supplementary Information

Below is the link to the electronic supplementary material.

Supplementary file1 (DOCX 436 kb)

## Data Availability

The datasets generated during and/or analyzed during the current study are available from the corresponding author on reasonable request.

## Footnotes

1 The AM1.5 G incident spectrum is commonly used to benchmark photovoltaics and represents incident sunlight on Earth’s surface at the geographical average latitude for the contiguous United States when the sun is 41.81 degrees above the horizon; it is a reasonable average amount of sunlight (averaged over a one year period) for any location in the United States, ignoring weather events (i.e. on a clear sunny day) [37, 56].

2 UVI is a measure of the erythemal UV radiation at Earth’s surface [57], and varies according to time of day, time of year, and location; measured UVI also takes account of cloud conditions. The World Health Organization advises that sun protection should be used when the UVI (measured or predicted) is 3 or higher [58].

## Code availability

N/A.
