# Supplementary material for: Zinc oxide-induced changes to sunscreen ingredient efficacy and toxicity under UV irradiation
Source: Photochem Photobiol Sci. 2021 Oct 14;20(10):1273–85. doi: 10.1007/s43630-021-00101-2 (PMC8550398; doi:10.1007/s43630-021-00101-2)
Supplement: Supplementary file 1 — Supplementary file1 (DOCX 436 kb) [file 43630_2021_101_MOESM1_ESM.docx]

**Supporting Information**

Zinc Oxide Induced Changes to Sunscreen Ingredient Efficacy and Toxicity Under UV Irradiation

Aurora L. Ginzburg,^a^ Richard S. Blackburn,^b^ Claudia Santillan,^c^ Lisa Truong,^c^ Robyn L. Tanguay,^c^ and James E. Hutchison^a^

^a^ Department of Chemistry and Biochemistry, University of Oregon, Eugene, Oregon 97403, United States, ^b^ Sustainable Materials Research Group, School of Design, University of Leeds, Leeds LS2 9JT, U.K., ^c^ Department of Environmental and Molecular Toxicology and the Sinnhuber Aquatic Research Laboratory, Oregon State University, Corvallis, Oregon 97333, United States.

Corresponding authors: [hutch@uoregon.edu](mailto:hutch@uoregon.edu) ; [r.s.blackburn@leeds.ac.uk](mailto:r.s.blackburn@leeds.ac.uk)

**Table of Contents**

| Section | Page |
| --- | --- |
| Supplemental UV-Vis Absorbance Spectra | 2 |
| UV-Filter Concentrations Used for *in vivo* Toxicity Assessments | 4 |
| UV-A Protection Factor Calculation | 5 |
| References | 5 |

**Supplemental UV-Vis Absorbance Spectra**

Samples were prepared to screen the stability of UV-filters in several compositions. In these screening experiments, the formulations were produced using volumetric methods, which were challenging due to the viscosity of some of the UV-filters, notably octocrylene. The approximate formulations of the active ingredients in Figures S1-S2 are: 2% avobenzone, 4% octisalate, 7% homosalate, and 5% octocrylene, which we confirmed by analysis of the optical absorbance in the spectra. Although the volumetric methods were sufficient for these screening experiments, where the goal was to establish a methodology, including the effect of adding non-active ingredients and the duration of UV exposure, subsequent, more quantitative formulas were produced using gravimetric approaches.


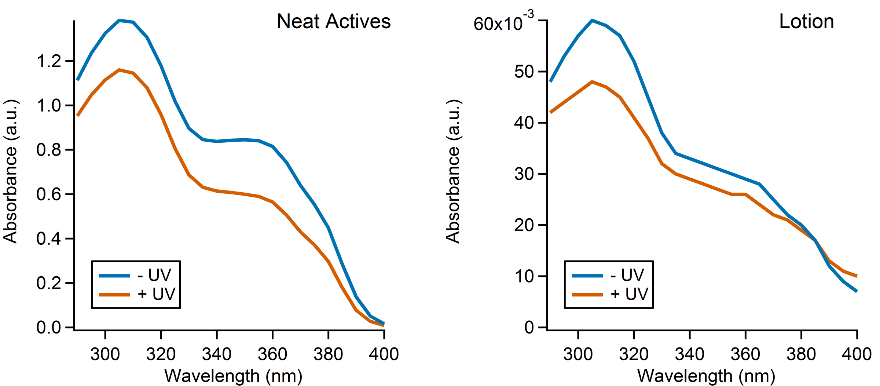


**Figure S1.** UV-Vis spectra showing similar results for studying just the active UV-filters versus the UV-filters plus lotion ingredients.

In these spectra, a moderately stable UV-filter mixture was formulated with only the active ingredients in DMSO (left) and with the actives plus lotion ingredients (right). The left spectrum was measured in 99:1 IPA/DMSO and the right spectrum was measured in 99:1 Water/DMSO. Both mixtures were exposed to UV irradation for 2 hrs and the lotion film was dissolved into DMSO following exposure. The results are similar despite the different solvent systems, the lotion degradation being highly dependent upon film thickness, and the small concentration of actives present in a thin film of lotion. This suggests that exposure of active ingredients in DMSO and measurement in IPA/DMSO is an informative way of simplifying the testing of UV-filter stability.


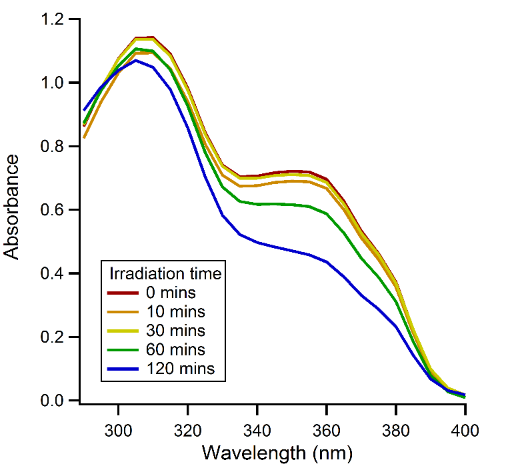


**Figure S2.** Irradation of a moderately stable UV-filter mixture over 2 hrs. Measured in 99:1 IPA/DMSO. The data show that photodegradation begins within 30 mins and continues occuring throughout the 2 hr exposure window.


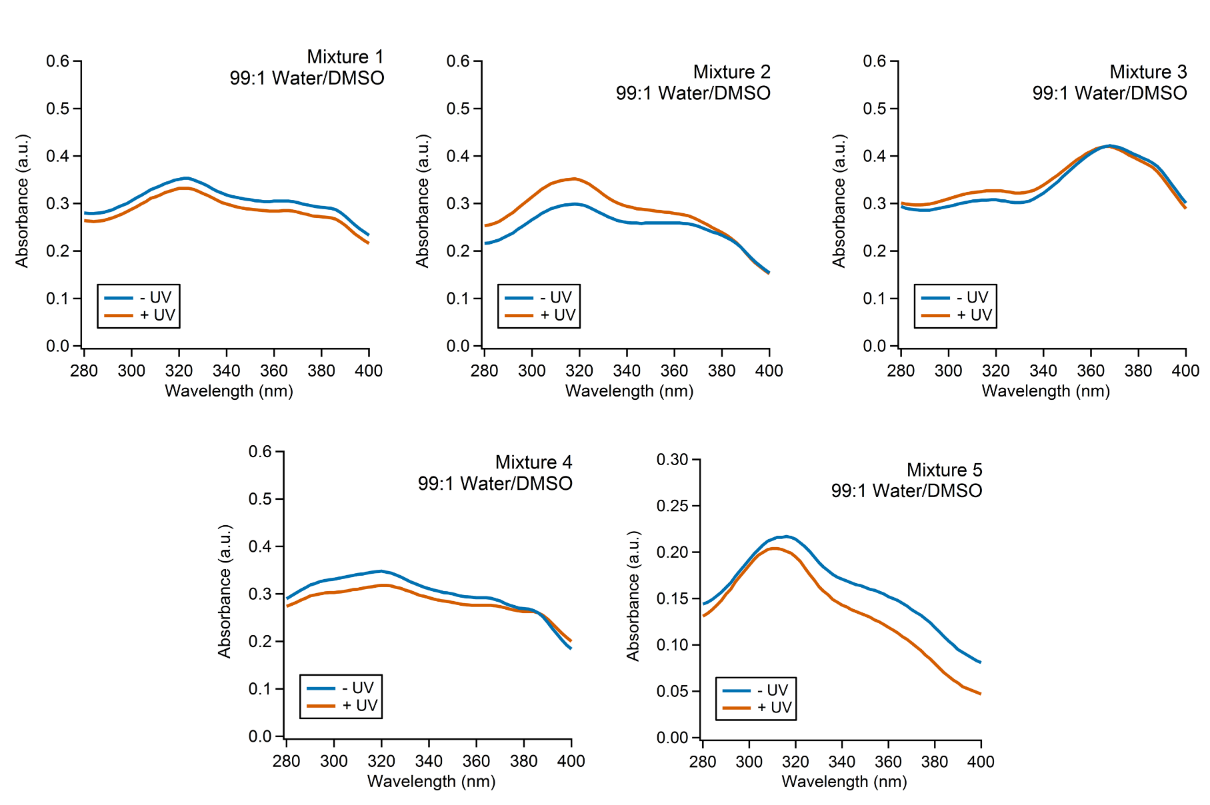


**Figure S3.** UV-Vis spectra showing photodegradation of mixtures 1-5. Measured in 99:1 Water/DMSO.


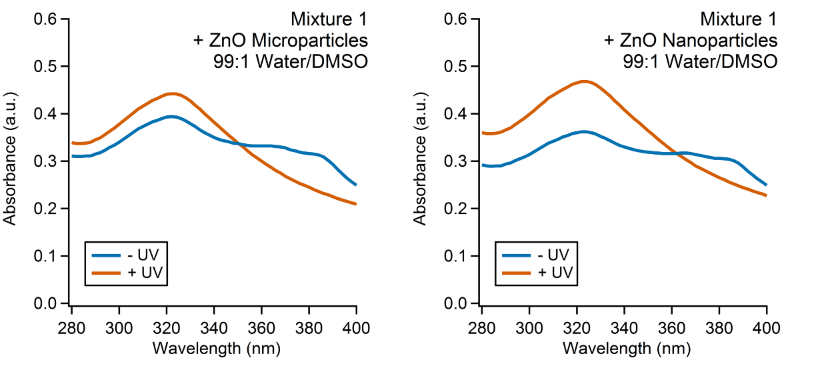


**Figure S4.** UV-Vis spectra showing photodegradation of mixture 1 + ZnO (nanoparticulate and microparticulate). Measured in 99:1 Water/DMSO.

**UV-Filter Concentrations Used for *in vivo* Toxicity Assessments**

**Table S1.** Final concentrations of UV-filters that fish were exposed to in diluted microwells.

| **Sample name** | **Final concentration**  **(% organic UV-filters)** | **Final concentration (%ZnO)** |
| --- | --- | --- |
| Mixture 1 - UV | 0.0014 | 0.0000 |
| Mixture 1 + UV | 0.0014 | 0.0000 |
| Mixture 2 - UV | 0.0015 | 0.0000 |
| Mixture 2 + UV | 0.0015 | 0.0000 |
| Mixture 3 - UV | 0.0030 | 0.0000 |
| Mixture 3 + UV | 0.0030 | 0.0000 |
| Mixture 4 - UV | 0.0015 | 0.0000 |
| Mixture 4 + UV | 0.0015 | 0.0000 |
| Mixture 5 - UV | 0.0015 | 0.0000 |
| Mixture 5 + UV | 0.0015 | 0.0000 |
| DMSO with UV | 0.0000 | 0.0000 |
| (Mixture 1 + ZnO microparticles) - UV | 0.0014 | 0.0005 |
| (Mixture 1 + ZnO microparticles) + UV | 0.0014 | 0.0005 |
| (Mixture 1 + ZnO nanoparticles) - UV | 0.0014 | 0.0005 |
| (Mixture 1 + ZnO nanoparticles) + UV | 0.0014 | 0.0005 |
| (ZnO microparticles) - UV | 0.0000 | 0.0005 |
| (ZnO microparticles) + UV | 0.0000 | 0.0005 |
| (ZnO nanoparticles) - UV | 0.0000 | 0.0005 |
| (ZnO nanoparticles) + UV | 0.0000 | 0.0005 |

**UV-A Protection Factor Calculation**

The UV-A protection factor (UVAPF) is calculated according to Equation S1:[1]

$\text{UVAPF}=\frac{\int_{\lambda=320}^{\lambda=400} P\left( \lambda\right)\times I\left( \lambda\right)\times d\lambda}{\int_{\lambda=320}^{\lambda=400} P\left( \lambda\right)\times I\left( \lambda\right)\times{10}^{-A\left( \lambda\right)}\times d\lambda}$ (S1)

where *P*(*λ*) is the persistent pigment darkening (PPD) action spectrum, the relative effects of individual spectral bands of an exposure source for a persistent pigment response [2, 3] and *I*(*λ*) is the spectral irradiance received from the UV-A source per unit wavelength, expressed in W m^-2^ nm^-1^; UV-A is 320 to 400 nm for PPD testing, and tabulated values for *P*(*λ*) and *I*(*λ*) are given in ISO 24443:2012 Annex C. *A*(*λ*) is the mean monochromatic absorbance of the test product layer; herein *A*(*λ*) values were measured in microplate with a 200 μL sample, corresponding to a pathlength of 0.61 cm. *A*(*λ*) values were calculated based on dilution factor of the sample. The method in ISO 24443:2012 applies a thin layer of sunscreen to a plate, so *A* values were converted to a modified pathlength of 10 μm, which is a typical thickness of the layer applied in the ISO method. *dλ* is the wavelength step (2 nm).

UVAPF before and after UV exposure was calculated from measured *A* values, and percent change in UVAPF calculated (Table S2).

**Table S2.** Percent Change in UV-A protection following UV exposure.

| **Sample name** | **UVAPF (% Change)** |
| --- | --- |
| Mixture 1 | -15.8 |
| Mixture 2 | -19.2 |
| Mixture 3 | 0.1 |
| Mixture 4 | 4.2 |
| Mixture 5 | -8.4 |
| Mixture 1 + ZnO microparticles | -91.8 |
| Mixture 1 + ZnO nanoparticles | -84.3 |

**References**

1. Technical Committee CW/217 Cosmetics (2012). ISO 24443:2012- *Determination of sunscreen UVA photoprotection in vitro.* The British Standards Institution.

2. Chardon, A.; Moyal, D.; Hourseau, C. (1997). *PPD action spectrum persistent pigment darkening response as a method for evaluation of Ultraviolet A protection assays in sunscreens – development, evaluation, and regulatory aspects* (pp. 559-582).

3. Moyal, D.; Chardon, A.; Kollias, N. (2000). UVA protection efficacy of sunscreens can be determined by the persistent pigment darkening (PPD) method (part 2). *Photodermatol Photoimmunol Photomed*, 16, 250–255
